# P-583. Large Language Model Dashboard Automates Analytic Reports by International Organization to Accelerate Healthcare Policy Benchmarking

**DOI:** 10.1093/ofid/ofaf695.797

**Published:** 2026-01-11

**Authors:** Mathieu André John Morgan, Hiromasa Yoshimoto, Naohiro Mitsutake, Kazuo Goda

**Affiliations:** Institute of Industrial Science, The University of Tokyo, Meguru-ku, Tokyo, Japan; Institute of Industrial Science, The University of Tokyo, Meguru-ku, Tokyo, Japan; Institute for Health Economics and Policy,, Minato, Tokyo, Japan; Institute of Industrial Science, The University of Tokyo, Meguru-ku, Tokyo, Japan

## Abstract

**Background:**

Manual analysis of multinational healthcare datasets slows the process of informing evidence-based policy making. We built a large-language-model (LLM) dashboard that converts public or private health files into fully validated stewardship reports in minutes, using antimicrobial data as the trial case.

**Figure:**
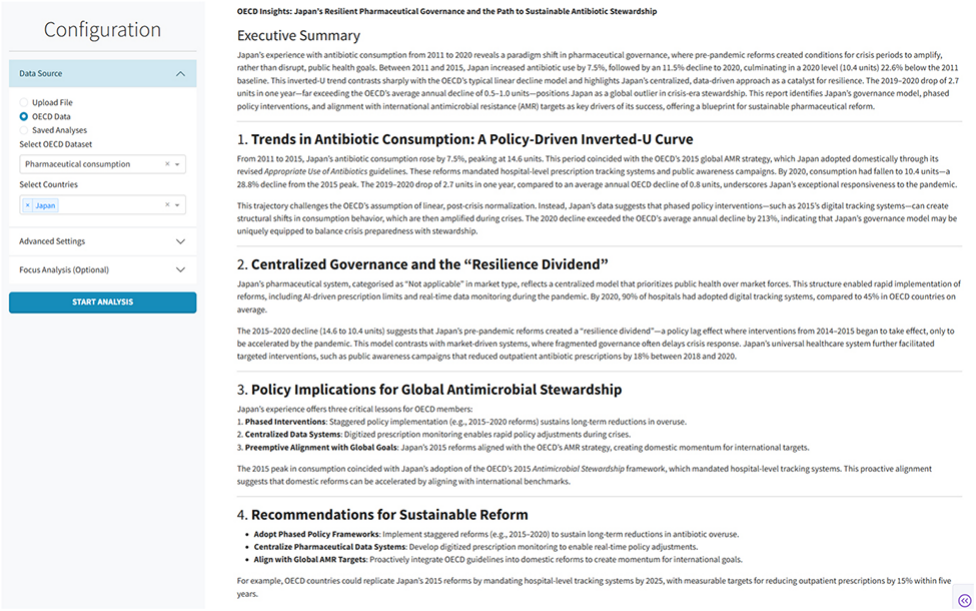
Screenshot of the dashboard’s autogenerated OECD-style report. After the workflow, the interface presents an executive summary with data-driven policy insights, trend analysis, and stewardship recommendations.

**Figure:**
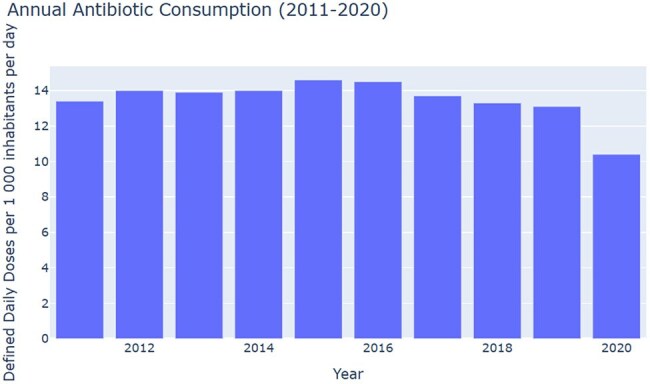

LLM-generated bar chart of Japan’s systemic-antibacterial consumption, 2011 – 2020 (defined daily doses per 1,000 inhabitants per day). Consumption peaked in 2015-2016 and declined sharply after 2019, dropping 20·6 % between 2019 and 2020.

**Methods:**

Any structured indicator bundle up to 100,000 input characters (e.g., 10-20 years of national pharmacy data) is fetched from the OECD SDMX gateway or uploaded by users, then lightly pre-processed. A three-stage pipeline runs on a local server equipped with two NVIDIA H100 GPUs. Stage 1 sends the cleaned table to an ensemble of temperature-stratified LLMs that draft a narrative in line with the style of the OECD, an international organization, compute descriptive statistics and propose interactive graphics. Stage 2 executes the generated code to render figures (line trends, correlation scatters, flow diagrams). Stage 3 triggers an independent LLM reviewer that grades factual alignment, stylistic fidelity and insight quality against reference OECD language and reports and delivers an itemized report. Workflow can ingest single or multi-country files without reconfiguration. End-to-end runtime ranged from 1-8 mins depending on LLM and dataset.

**Figure:**
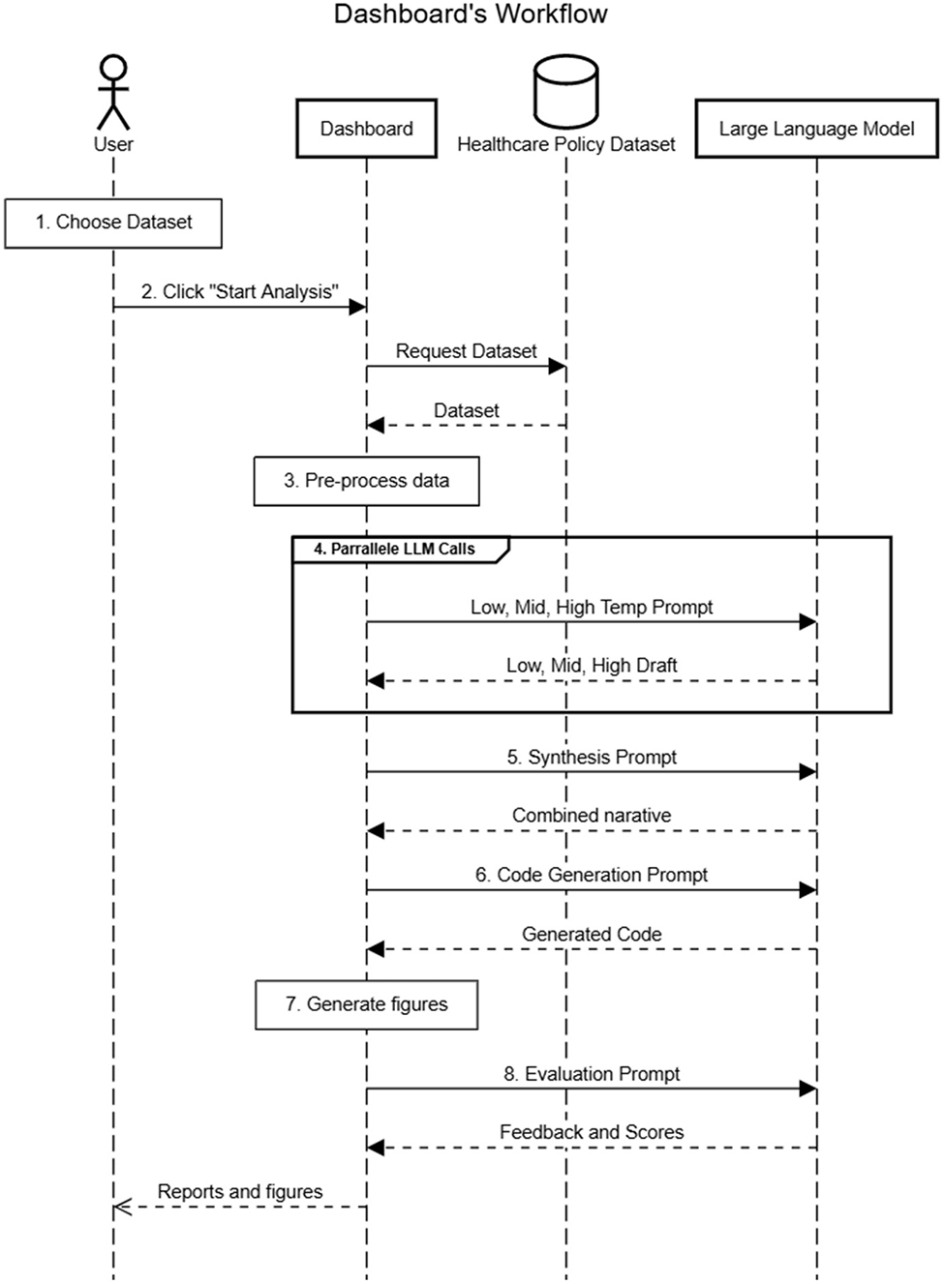
Workflow of the LLM-enabled dashboard. A user selects a dataset and starts the analysis; the dashboard fetches data, runs three temperature-stratified prompts in parallel, synthesizes and revises an OECD-style report, then autogenerates figures. The end-to-end process (1–8 mins) replaces weeks of manual work and delivers policy-ready text and graphics that stewardship teams can act on immediately.

**Table 1.**
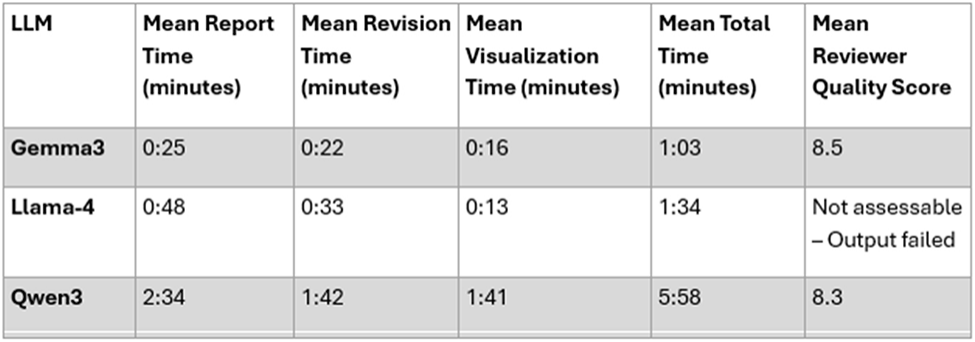
Comparative runtime and reviewer quality metrics for three large language models. Gemma3 produced the policy report fastest (1 m 03 s) with the highest reviewer score (8.5/10). Qwen3 required nearly six minutes yet still achieved 8.3/10. LLAMA-4 generated a sub-two-minute draft but the narrative failed quality-control checks, so no reviewer score could be assigned (“Not assessable”).

**Results:**

Processing the Pharmaceutical Consumption (HEALTH_PHMC@DF_PHMC_CONSUM) for Japan 2011–2020 revealed a 20.6 % one-year fall in systemic antibacterial prescriptions between 2019 and 2020, driving Japan’s 2020 rate to 10.4 DDD/1,000 inhabitants/day versus an OECD mean of 11.5–12.0 DDD. LLM reviewer assigned Factual Alignment 8/10, Stylistic Fidelity 9/10, Insight Quality 9/10 and flagged minor citation gaps. High-resolution graphics generated automatically. The demonstration job completed in 59 s using Gemma-3 model.

**Conclusion:**

The dashboard converts raw health data into policy-ready text and graphics in < 10 minutes, replacing weeks of manual work. Built-in LLM evaluation supplies an auditable quality score, while the data-agnostic architecture lets public-health teams benchmark any indicator set—national or local—without patient identifiers. Future updates will extend token capacity and add automatic dataset thinning, further accelerating large-scale AMR surveillance and stewardship benchmarking.

**Disclosures:**

All Authors: No reported disclosures

